# Utilizing a Wristband Sensor to Measure the Stress Level for People with Dementia

**DOI:** 10.3390/s16121989

**Published:** 2016-11-24

**Authors:** Basel Kikhia, Thanos G. Stavropoulos, Stelios Andreadis, Niklas Karvonen, Ioannis Kompatsiaris, Stefan Sävenstedt, Marten Pijl, Catharina Melander

**Affiliations:** 1 Department of Health Sciences, Luleå University of Technology, 97187 Luleå, Sweden; stefan.savenstedt@ltu.se (S.S.); catharina.melander@ltu.se (C.M.); 2 Information Technologies Institute, Centre for Research & Technology Hellas, 57001 Thessaloniki, Greece; athstavr@iti.gr (T.G.S.); andreadisst@iti.gr (S.A.); ikom@iti.gr (I.K.); 3 Department of Computer Science, Electrical and Space Engineering, Luleå University of Technology, 97187 Luleå, Sweden; niklas.karvonen@ltu.se; 4 Personal Health Solutions, Philips Research, 5656 AE Eindhoven, The Netherlands; marten.pijl@philips.com

**Keywords:** clinical assessment, nursing homes, sensors, dementia, stress monitoring

## Abstract

Stress is a common problem that affects most people with dementia and their caregivers. Stress symptoms for people with dementia are often measured by answering a checklist of questions by the clinical staff who work closely with the person with the dementia. This process requires a lot of effort with continuous observation of the person with dementia over the long term. This article investigates the effectiveness of using a straightforward method, based on a single wristband sensor to classify events of “Stressed” and “Not stressed” for people with dementia. The presented system calculates the stress level as an integer value from zero to five, providing clinical information of behavioral patterns to the clinical staff. Thirty staff members participated in this experiment, together with six residents suffering from dementia, from two nursing homes. The residents were equipped with the wristband sensor during the day, and the staff were writing observation notes during the experiment to serve as ground truth. Experimental evaluation showed relationships between staff observations and sensor analysis, while stress level thresholds adjusted to each individual can serve different scenarios.

## 1. Introduction

Stress is a common problem that is experienced by people with dementia and their caregivers [1]. There are different symptoms that people with dementia show when they are stressed such as apathy, aggression, sleep disturbances, wandering, and depression [2]. In general, Behavioral and Psychological Symptoms of Dementia, labeled as (BPSD), affect more than 90% of people who suffer from dementia [3]. BPSD are divided into symptoms of agitation, delusions, euphoria, hallucinations, apathy, depression, aberrant motor behavior, irritability, eating problems and sleep problems [4]. BPSD affect not only people with dementia, but also caregivers as they are closely connected to the person with dementia on a daily basis. Statistics show that 74% of caregivers were concerned about maintaining their own health since becoming a caregiver. In addition, 40% of caregivers reported that the emotional stress of their role is high or very high [5].

Understanding BPSD and the changes of behavior of the person with dementia helps in assessing the development of the disease. Clinical staff will also be able to monitor the treatment of behavioral symptoms, and in planning their clinical interventions to reduce the BPSD and improve the status of the person with dementia. One key factor when assessing BPSD is to identify moments when the person with dementia is stressed, and then search for the possible causes of these stress episodes to reduce it. Stress and anxiety can be a result of social isolation, unfamiliar surroundings, environmental factors such as light and noise, mental confusion, physical inactivity, etc. [6,7].

The common method in assessing BPSD in nursing homes is to rely on the staff observations in defining different symptoms that the person with dementia has. This is done with the help of the Neuropsychiatric Inventory-Nursing Home version (NPI-NH) instrument [8]. The instrument provides a list of questions about the person with dementia that covers the BPSD domains. Answering the questions by the caregiver will result in a score for each domain and an overall score that gives hints about the status of the person with dementia. From this assessment, the clinical staff can locate possible problems and plan their clinical interventions to reduce the BPSD. The NPI-NH instrument, however, requires a lot of effort with continuous observation of the resident over long periods of time in order for the caregiver to get an understanding of the behavior [9]. The whole process of using the NPI-NH instrument is thus both complicated and time-consuming, which makes it impractical to use for the clinical staff.

To help caregivers and clinical staff understand the behavior of the person with dementia, and to simplify the assessment of BPSD, this work presents a sensor-based system that measures the stress level of people with dementia. The system detects stress patterns, which will provide objective clinical information that helps caregivers in planning clinical interventions to reduce stress and aggression for people with dementia. Reducing stress and aggression for people with dementia will also result in reducing the stress for the caregivers, and for other people in their environment. These other people include residents in the nursing home, or family members living with the person with dementia at home. The system thus could contribute to improving the status of the person with dementia, and in the nursing homes it could significantly improve the quality of care and reduce the care needs, and further reduce costs.

The system relies on a single wristband that is worn by the person with dementia during the day. The use of a single sensor is important to simplify the system, so it does not create any burden for the caregivers, or for the person with dementia [10]. Having multiple sensors will increase the complexity of the monitoring system and make it more cumbersome for the users [11]. While many advanced machine learning methods have been used to measure stress using multiple information sources [12], we aim for a more straightforward method based on such limited input. The data from the wristband is transferred and processed in an online service that calculates the stress level that the person with dementia had. This data is presented to the caregivers and the clinical staff in a timeline, including a single-day view and a multi-day view. The system also marks patterns of high stress over a longer period, so the clinical staff can observe those patterns and identify regular stress episodes that the user experiences.

The usability and the effectiveness of the above-described sensor-based system were explored by the authors in a previous study [4]. In this article, the authors extend their previous work by validating the proposed system. Episodes of stress that are detected by the system are compared to observation notes written by the caregivers to evaluate the accuracy of the system. The work investigates if it is feasible to rely on a wristband band sensor for detecting stress episodes that people with dementia experience. The remainder of this article is organized as follows: Section 2 presents a review of the related work. In Section 3, the design of the study within this work is discussed. The design section includes a presentation of the wristband and the data collection protocol. Data processing using the proposed system is presented in Section 4. The evaluation results from the study as well as clinical user interfaces are discussed in Section 5. Finally, conclusions and future works are presented in Section 6.

## 2. Related Work

Many works have focused on other aspects of wearable sensors with success. Gjoreski et al. [13] examined fall detection from accelerometer measurements on wearable sensors. Such insights can be useful to provide real-time alerts for caregivers to intervene. On the other hand, our work focuses on treatment over a longer period and quality of life by reducing and intervening in stress by using wearables. Therefore, this section considers the state-of-the-art in stress detection rather than activity recognition and fall detection.

Many studies suggested the use of physiological signals to detect stress and measure emotions of people, such as Skin Conductance [14], Blood Volume Pulse (BVP) [15] and Electroencephalography (EGG) [16]. Kirschbaum et al. [17] measured the cortisol in saliva to investigate the hypothalamus-pituitary-adrenal (HPA) axis activity. The HPA is usually activated when the person is experiencing a stressful situation, and it is easily measurable in saliva, urine, and blood. Blood pressure can be also used to give information of the stress level that people have, and it can be measured by a vital signs monitor. Lupien [18] explained in her book that two numbers appear when measuring the blood pressure. The first one represents the systolic pressure, and the second one represents the diastolic pressure. These numbers could be a source for detecting a stressful moment for the person. However, measuring the cortisol in saliva and blood pressure cannot be done unobtrusively with the technology available today. This makes it unsuitable for continuous observations of a person over longer periods of time in everyday life.

Villarejo et al. [19] introduced a stress sensor based on Galvanic Skin Response (GSR), also known as skin conductance, and controlled by ZigBee. The sensor has two electrodes that are placed on the fingers, and it sends the data via ZigBee to a coordinator that forwards it to a computer. Sixteen adults participated in the experiment and the results showed a success rate of detecting stress by 76.56%. Bakker et al. [20] proposed the use of a watch-style stress measurement device that captures stress-related physiological signs. The aim of the framework is to manage stress at work by making the workers aware of the past, current or expected stress. Their watch had two electrodes that require skin contact in order to produce a reliable signal that is used to give two labels: “stressed” or “not stressed”. The results indicated that additional data to GSR is needed to make a detection of the stress level, as there are varieties of patterns in the GSR data.

Perala et al. [21] presented a study to measure the soldiers’ stress in military using GSR. The authors in [21] compared the stress results from the GSR method with the traditional survey method. The aim was to verify if GSR is actually measuring stress. GSR data was collected by using a small, lightweight, unobtrusive body monitor armband. The armband was worn by the soldiers on the back of the upper arm during the day. The results indicate that GSR is a promising continuous measure of stress that does not entirely rely on biased self-reporting. Another experiment was conducted by Hernandez et al. [22] to measure the stress of call center employees after each call they receive using a wrist skin conductance sensor. Nine employees participated in the experiment, who wore the sensor and made self-report ratings at the end of each call. The reports were used to verify if the skin conductance results were accurate, and the results showed 78.03% accuracy in detecting stressful moments.

The aforementioned works, however, focused on healthy adults as a target group, and not on people with medical problems, e.g., dementia. In addition, not many studies had a continuous observation of the user, as they investigated methods to give an indication of the stress level when performing a specific test for the physiological signals. Our work performs an experiment with people with dementia in the natural settings of nursing homes. The aim is to have a continuous observation of the user and test the GSR response on this target group in uncontrolled environment.

A very recent study by Sarker et al. [23] uses sophisticated time-series mining to predict significant stress episodes for just-in-time interventions. For this, it utilizes a flexible band worn around the chest, providing rich data (accelerometer, electrocardiograph, and respiration). It also examines several cofounding factors, such as the varying recovery time and physical activity. The need for personalization for both stress measurement and intervention is showcased, as the two most stressed participants are on average twice as stressed as the two least stressed ones. On the other hand, our work aims for long-term monitoring and interventions in nursing homes, instead of just-in-time ones. In this framework, it utilizes a single comfortable wrist-worn sensor for simplicity, which provides less input, and thus employs less sophisticated processing.

Another study utilizes various sensors and information on smartphones to assess stress in school students [12]. Information input includes activity (smartphone accelerometer), Wi-Fi usage, light levels, ambient audio such as conversations or noise, battery charge and call logs. Questionnaires were filled out by the students to self-report severe, mild, or no stress. Learning algorithms were employed using the above information as features along with school schedule information (such as midterm exams). Building a model for each student using the rest of the students yielded poor results (up to 43% accuracy). Clustering students of similar traits demonstrated the same performance. Both of these results led to the need for a personalized model, possibly due to high subjectivity of perceived stress, i.e., different people appraising the severity of the same negative effects differently, according to diverse personalities, coping resources and support [24]. Indeed, higher accuracy (60%) was achieved when using the initial data for each student as a calibration (test) phase. To some extent, our method is in-line with this approach, calibrating the model with the initial data for each user. Notably, many dissimilarities do exist when targeting the elderly instead of students, such as logs from the use of technology (battery, calls, and Wi-Fi), as well as the school schedule information (e.g., midterms).

Focusing on measuring stress in the dementia domain, Rodney [25] did a study to explore the relationship between the stress of the caregivers and the stress/aggression that people with dementia show. The author measured the stress of people with dementia using a checklist of questions that was introduced by Mackay et al. [26]. The checklist was given to the caregivers in a self-report format, and they were instructed to answer based on their experience with the person with dementia. The checklist contains 19 adjectives such as “tense” and “peaceful”, and the answers yield a maximum score of 57. This score was used to give an estimation of the level of stress/aggression of the person with dementia. The results of the study indicated that the increased stress of the caregivers is strongly related to the stress/aggression level of people with dementia.

Another study was conducted by Vedhara et al. [27] to investigate the effect of the stress on the immune system for people with dementia and their caregivers. The stress level of people with dementia was measured using the Global Measure of Perceived Stress scale [28]. This scale consists of 14 items that measure if a moment of the person’s life is considered stressful. The scale also has queries about an experienced stress during the day of the person. These studies used the traditional method of a list of questions that the caregivers should answer to have an understanding of the mental state of the person with dementia. The work presented in this article relies on a sensor to measure the stress, so it eliminates the efforts and the time that is consumed by the staff in answering the questions.

There are very few studies that focused on the use of sensors to measure the stress for people with dementia. Algase et al. [29] used the step watch to measure the wandering behavior of people with dementia. The wandering activity was used as an indication of stress for the person. There were, however, no studies that illustrated the use of sensor technology for people with dementia in nursing homes to measure the stress level. We are not familiar with a similar study to the one presented in this article, as we are focusing on measuring stress for a special target group in a natural living setting. Table 1 compares our work to the related work presented before.

## 3. Design

The study was conducted in two nursing homes in the northern part of Sweden. The staff members in the nursing homes are familiar with the NPI-NH instrument [8], and they usually use a computerized system for manually registering BPSD domains and visualizing them long-term. This manual registration is part of a national program in Sweden for improving the quality of care of people with BPSD [30]. Residents of the nursing homes live in their own apartments, and the study was carried out in natural settings. Trained research staff, both technical experts and experts in dementia care, set up the test including training and supervising the staff in the nursing homes. The pilot involved thirty staff members and six participants with dementia. The participants are diagnosed with Alzheimer’s disease, and they were recruited to take part in the data collection based on their BPSD problems. Problems include, for example, sleeping problems, anxiety, difficulty with orientation, disturbance by other residents, agitation, and wandering.

### 3.1. Deployment

**Figure 1:**
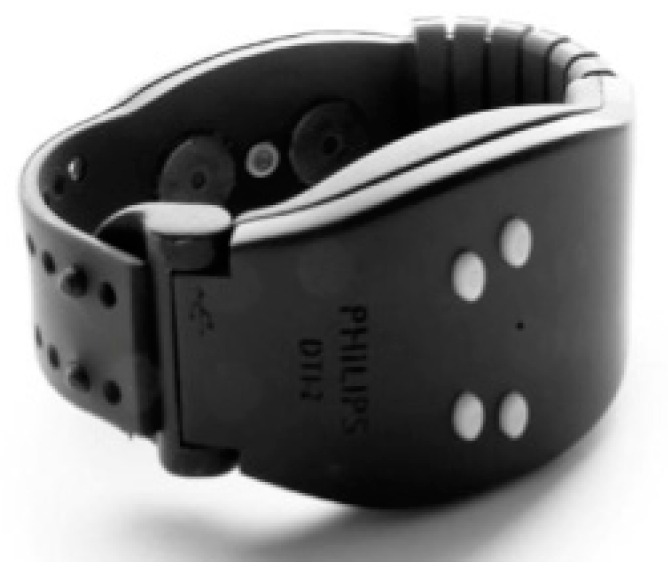
Philips DTI-2 wristband sensor.

The platform, called DemaWare@NH, was explained in details by the authors in [4]. The DemaWare@NH system highlights the most common behavioral and psychological problems of people with dementia that are related to stress [1]. It consists of a wristband sensor and an online service that processes the sensor data and visualizes the assessment results. The wristband sensor is a wearable skin sensor that is worn at the wrist by the participant, and activated by the staff. The wristband collects a number of different parameters, typically GSR (skin conductance), accelerometers (for capturing motion), skin temperature, environment temperature and environment light. Figure 1 shows the wristband sensor that was deployed during the pilot, namely the Philips sensor DTI-2 (Philips Research, Eindhoven, The Netherlands) [31].

### 3.2. Data Collection

#### 3.2.1. Sensors

The DTI-2 sensor was activated and put on the participant’s wrist by the staff when the participant was awake, and was put on a charger when the participant was sleeping. The aim was to observe the stress level of the participant when the user was awake during the day or the night. Each participant had the sensor for a period of two months. The researchers collected the data from the sensor on a weekly basis and processed it using the platform.

#### 3.2.2. Observation Notes

**Figure 2:**
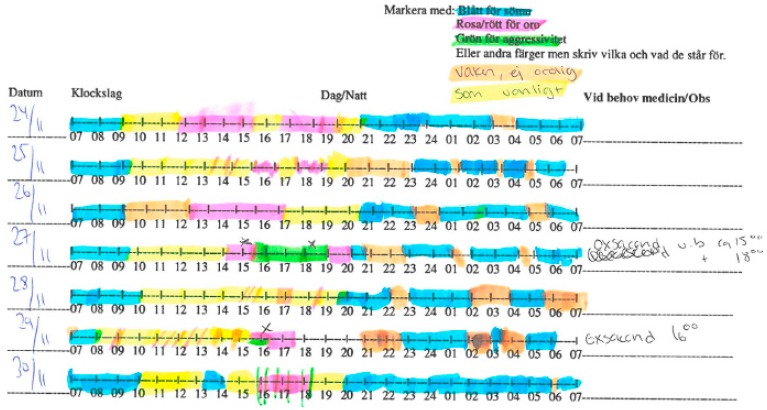
An observation note filled in by the staff for a participant over a week.

The staff were provided with observation notes to indicate the status of the person with dementia based on their own observations. The notes had a timeline of 24 h and four categories, including Sleeping, Aggression, Stress, and Normal. The staff were asked to mark moments of the day based on the categories using specific colors for each one. For instance, an hour of stress for the participant will be marked on the timeline with red, while the periods of sleep will be marked with blue. An example of the observation notes for a participant is shown in Figure 2. As the data collection was conducted in natural settings, there were moments that the staff forgot to mark the observation notes. The notes, however, provided a wealth of information for the researchers to compare with the system’s results after processing the sensor’s data.

**Figure 3:**
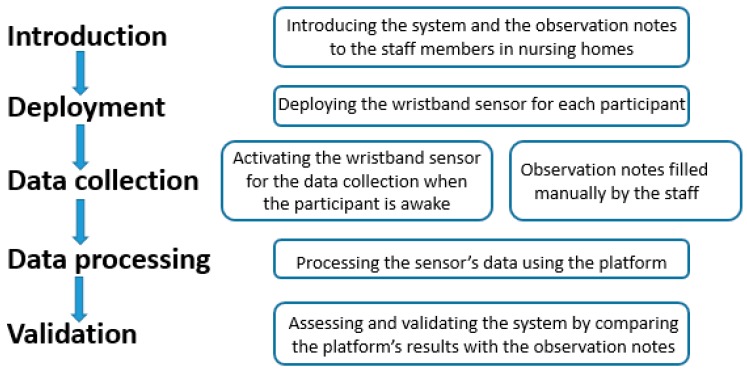
The overall design of the study.

Figure 3 summarizes the overall design of the study.

## 4. Data Processing

The data of the DTI-2 was processed by the researchers for each participant on a weekly basis. Data was analyzed by the platform based on the GSR measurements. GSR is one of the known indicators for stress and other emotional states, such as agitation or aggression [32]. Stress is often accompanied by a physiological response, which results in an increase of GSR, which can be measured between two electrodes placed on the skin, in nano-Siemens (nS). The signal entails two components: the rapidly-changing skin conductance response, which corresponds to short-term external stimuli, and skin conductance changing more slowly (after several seconds or more). The latter is the signal of interest here, as it reflects longer-term emotional changes regardless of external stimuli.

The GSR signal is often noisy, as it contains many artifacts due to movement, short-term spikes and noise due to various causes. Another drawback is the high variability of the measurements between individuals, as apart from arousal and stress, GSR is also influenced by other physiological processes and external factors such as ambient temperature. Thus, a baseline needs to be established per individual after a short period of observation. In this approach, we create a baseline to statistically classify each measurement as high or low stress. Initially, the method applies filtering to clear out artifacts and noise from the signal. The filtering steps are given below:

- No-contact zero values: the signal often contains instances of values equal to zero or very close to zero. These values correspond to moments that the sensor lost skin contact, either intentionally or unintentionally, due to movement. Therefore, if 90% of values within a 5 s window do not exceed the lower bound threshold (experimentally found to be 0.001 nS), then they are removed.
- Removing Spikes: generally, GSR levels were experimentally found to be changing no more than 20% when increasing and 10% when decreasing, each second. A moving one-second median filter is used for an initial interpolation to even out the signal. Outliers exceeding those values are considered noise.

These filtering methods mostly remove noisy intervals from the signal, such as the initial period of sensor calibration and final periods of moving and taking the sensor off.

- Obtain the long-term skin conductance component: in order to obtain the slowly changing (after several seconds) component of skin conductance, a low-pass filter is applied, in the form of a sliding window filter, with a window size of one minute, accepting values when at least 60% of the window is not noise.
- Filling the noise gaps: linear interpolation is used to reconstruct the signal, filling the gaps due to noise. However, the values generated in this step are not considered when setting a baseline or categorizing stress.

After the signal has been cleared, the stress measurement method entails baseline construction and actual classification:

- Baseline Construction: a baseline is needed for each individual in order to achieve a personalized stress interpretation. The baseline is kept in the form of a 600-bin histogram storing the frequency of the appearance of each skin conductance level. This frequency is measured over the entire signal. If a previous histogram entry exists, the new histogram is merged into it.
- Stress level measurement: The method follows state-of-the-art guidelines to determine the lowest, i.e., resting stress level [32]. Specifically, it assumes that a five-minute window that the person is at rest can be found, for which the maximum skin conductance level within it is the smallest of all other maximum values of five-minute windows in the signal (excluding noise). This lowest maximum is called the zero level, i.e., ‘10’, and serves as the lowest level of stress encountered in the recordings. Combined with the skin conductance level histogram, l0 is stored as part of the baseline.

The next step is to find a reasonable cut-off point for high stress, i.e., the ‘l5’ level, as the algorithm considers five distinct stress levels. The l5 level of skin conductance is selected using the baseline histogram as the first level (greater than the mean), for which no values in the baseline have been observed. To avoid setting a value of l5 that is too low, less than 40% of observed levels should be greater than the chosen value.

The remaining levels are distributed equally, according to the formulae below:

(1) $$\begin{array}{l} \delta =\frac{l5-l0}{4.5}\\ l1=l0+\frac{1}{2}\delta \\ l2=l1+\delta \\ l3=l2+\delta \\ l4=l3+\delta \end{array}$$

Finally, a given value is classified within each stress level boundary. Notably, a single value is not selected to serve as a threshold. As a result, the algorithm does not actually highlight events of stress, but rather stops at defining a stress level from one to five for each time segment. Indeed, as thresholds can yield different outcomes, this allows for further tailoring the method at later stages, e.g., by setting a threshold at the GUI. The evaluation section shows an investigation of how threshold values affect performance.

## 5. Evaluation and Results

The goal of this experimental evaluation is to show how well the sensor analysis methods, as described in Section 4, manage to align to clinical observation notes. First of all, we start with a description of the data set collected and utilized in this study. Then, the process of evaluation is described. To begin with, clinical notes and sensor data are incompatible with one another, both as modalities and temporal entries. Therefore, we establish a common information schema and mapping methods to evaluate stress predictions across corresponding clinical notes.

In detail, the evaluation framework is established as follows:

- A common information schema is devised as a common base between both notes and measurements.
- Clinical notes are translated/mapped upon this schema.
- Stress measurements from sensor data analysis are mapped and translated to the information schema using a threshold.
- Corresponding measurements are evaluated across corresponding clinical notes using standard metrics, for various threshold settings and individuals.

### 5.1. Data Set

The evaluation process utilizes a collected dataset from six individuals living in the nursing home, along with ground truth obtained from clinical notes. In detail, the dataset contains skin conductivity measurements per minute, while clinical notes mention the segments of stress incidents for the involved individuals. While the former type of data is quite commonly found online with the emergence of wearables and the Internet of Things (IoT), the latter is quite rare and hard to obtain, as it involves a clinical, ethically-approved and well-defined process. Using this dataset as the only benchmarking resource, at least to the best of our knowledge, prohibits comparisons with existing techniques at this stage.

**Figure 4:**
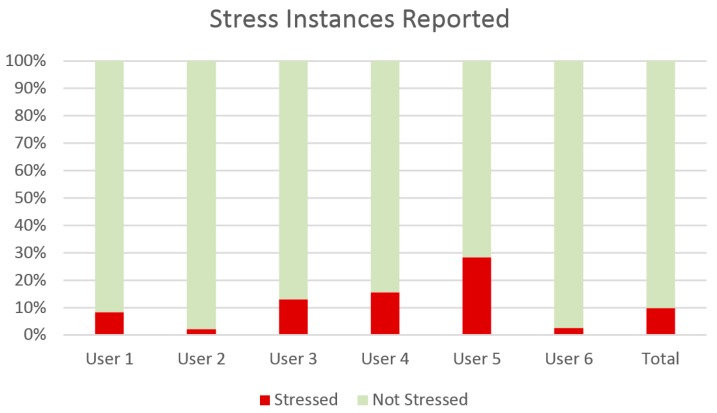
Class distribution in the dataset as a percentage of instances of stress reported in observation notes out of the total hours logged.

Table 2 provides some insights to the data set collected and used in this evaluation. In detail, it shows positive, negative, and total instances for each user, treated as a separate set. Each instance corresponds to an hour-long observation marked as either “Stressed” (Positive) or “Not Stressed” (Negative) by clinicians, reaching up to more than 2400 instances. However, the percentage of positive instances to the total is in most cases quite low. This is shown clearly on Figure 4, which displays the percentage of stressed versus not stressed observation instances, total and per person.

### 5.2. Information Schema

While the skin conductivity processing method provides stress measurements, the evaluation process provides a series of transformation steps for them and the clinical notes alike in order to synchronize them and yield performance metrics. Initially, clinical notes in a piece of paper, as shown in Figure 2, have to be transformed into a machine-interpretable format and also strictly defined to serve as ground truth. Therefore, we defined a structured schema for clinical observations to be mapped to ground truth. Clinical notes do not necessarily account for the exact monitoring period, but, on the contrary, overlap it. Hence, the schema should specify whether an incident refers to ‘Stressed’, ‘Not Stressed’ or no observation (lacking notes). The same schema should similarly be able to accommodate the observations derived from sensor measurements. The resulting schema is defined as shown in Table 3:

In this schema, each event is characterized by a starting and an ending timestamp, and a unique (anonymous) person ID. The event type is either a ‘Stressed’ or ‘Not Stressed’ label while the information provider field is either the ‘Clinical Notes’ or the ‘Sensor’ analysis component, covering effectively all requirements for evaluation.

### 5.3. Mapping Clinical Notes to Information Schema

In order to transform handwritten notes to the information schema for ground truth, clinical staff used simple spreadsheets (Excel), as a human-friendly authoring tool. The notes are divided into hourly segments. The researchers used Excel to insert one row for each instance of consecutive hours with the same observation (type), either stressed or not stressed. Following the given example, Sleeping and Normal are considered no stress observation, while Aggression and Stress are considered simply stress. The resulting Comma Separated Values (CSV) files were generated from the spreadsheets and imported into the evaluation tool’s database to serve as ground truth.

### 5.4. Mapping Stress Measurements to Information Schema

In general, stress measurements extracted from sensor measurements processed by skin conductivity analysis also have to align to the common information schema in terms of structure. For this reason, the outcomes from the analysis have to either signify stressed or not stressed instead of providing stress level as a numerical value. Since the values are integers between one and five, they are actually investigated in this method as a parameter whose value could yield the best results. This investigation is presented further in this section.

Apart from aligning to the schema, the information between sensors and ground truth should also align temporally in order to perform direct evaluation comparisons. For this purpose, analysis outcomes are aggregated and rounded to provide a single value per hour. Each hour-long segment, furthermore, begins at minute zero and ends accordingly as provided in the clinical notes. To aggregate the values, the method averages values within each segment.

### 5.5. Metrics

After aligning actual stress incidents from ground truth and predicted outcomes, they can be directly compared to measure the following attributes: *TP* (true positive), *FP* (false positive), *TN* (true negative), and *FN* (false negative). In turn, these attributes can be combined to provide a comprehensive set of metrics. The method considers the following metrics, as established in the field of information retrieval and binary classification:

(2) $$Precision=\frac{TP}{TP+FP},$$

(3) $$Recall=\frac{TP}{TP+FN},$$

(4) $$Accuracy=\frac{TP+TN}{TP+FP+FN+TN},$$

### 5.6. Results

The sensor recordings from the six participants added up to 142 h across 37 (calendar) days, with corresponding clinical notes of an equal number of (full) days. The goal of this study is to evaluate the effectiveness of classifying events i.e., time segments as either ‘stressed’ or ‘not stressed’. To do so, we treated time interval (sampling rate) and stress value threshold as parameters to be optimized. Experimentation with the time interval parameter, from one minute, thirty minutes, an hour, two hours etc., resulted in a clear, monotonous optimization of all metrics when sampling events per hour. Due to this result being straightforward, we do not thoroughly present performance for other sampling rates, but rather consider the one hour period as a given for the rest of this study.

The second parameter to optimize is the numeric threshold above which we consider stress level measurements to signify a stress event. As presented previously, the current stress algorithm outputs stress level as an integer value from zero to five. Indeed, this is suitable for cases where thresholds have to be adjusted to certain conditions, e.g., environmental or physiological. In order to find the optimal stress threshold for this particular study, we performed the evaluation for all metrics and all participants for each distinct integer value from zero to five. For each value, an interval was marked with the ‘stressed’ label if the average value within the interval was larger or equal to that. Note that for a threshold equal to zero, all intervals are considered as stress.

Table 4 summarizes the results for all metrics and for each distinct stress level threshold. Generally, the outcome helps support the choice of using a different threshold according to the situation. As expected, raising the threshold causes fewer events to be classified as stress, due to harder criteria. This, in turn, increases precision, i.e., the percentage of true positive events within all positive events. Still, it peaks at level four, dropping low at level five. This goes to show that perceived (observed) stress corresponds with middle-to-high physical responses and not high ones. Meanwhile, recall drops monotonously, as many actual stress events are filtered out by the increased threshold, i.e., fewer true positive events are found out of the total actual stress events. Accuracy, much like precision, is also increased as the threshold increases. This is due to more accurately identifying true negatives along with true positives. On the other hand, the F-measure clearly does not favor extremes, neither the lowest nor the highest value, offering some balance. Combined with precision peaking before level five, a threshold of three to four offers a well-balanced solution.

**Figure 5:**
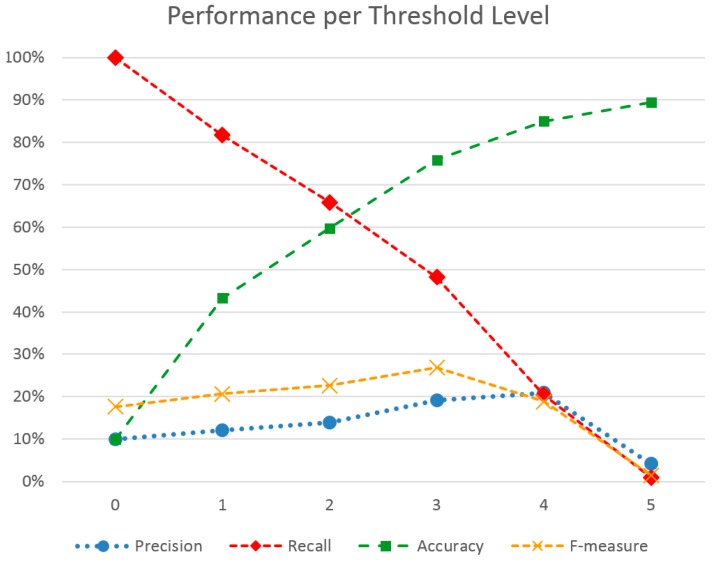
Performance metrics across different stress level thresholds.

Figure 5 shows the continuous change of metrics alongside the threshold. As F-measure also confirms, an optimal threshold for increased precision, accuracy and decent recall is level 3. However, this does not mean that this threshold should be used in all cases. Presenting all the metrics in this study gives a holistic picture of the quality each threshold gives. This allows clinicians to pre-select a threshold according to scenario. For example, if interested in seeing all stress events while tolerating false positives, a low threshold can be chosen, such as three. On the contrary, if interested in receiving severe events only, or else getting a high percentage of actual stress events amongst the selected ones, a high value such as four or five would suit the requirements better.

**Figure 6:**
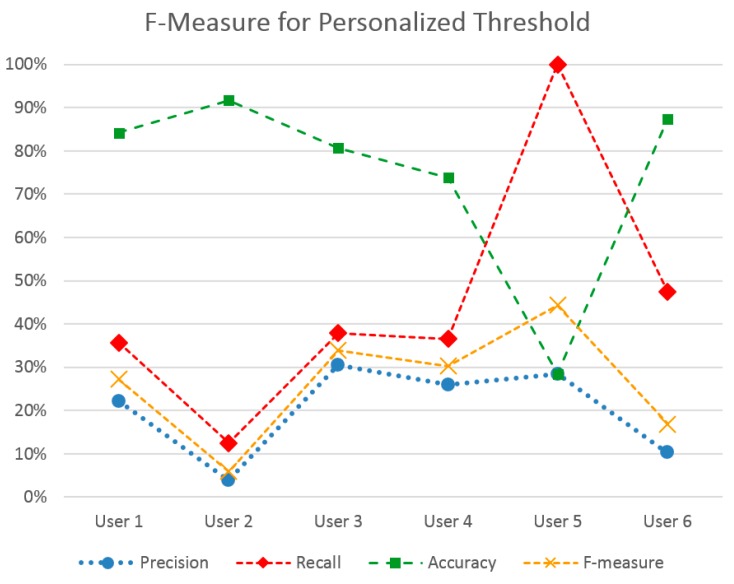
Performance metrics of the personalized threshold method to optimize F-Measure, per each user.

These outcomes have led to a further personalized method, where a stress level threshold is previously set. In this case, the threshold to optimize the F-measure metric was chosen for each user, yielding the outcomes shown in Figure 6. Indeed, F-measure score has been increased for most users. The best performing user-set is User 5, which also holds the highest ratio of positive to negative instances in his set. The lowest F-measure is met in Users 2 and 6, which, at the same time, hold a quite low positive to negative instance ratio, hinting at how the two factors might be linked. Indeed, the method utilizes the initial measurements of a user to classify stress events and not the entire set. This means that adequate stress events should have been noted in this initial measurement period.

### 5.7. Clinical View

Based on the above results, two methods for the clinicians to monitor individuals are apparent: the general method, where no threshold for stress level is set or known, and the personalized method, where the clinician picks an optimized stress level threshold for the scenario or individual. This was done to provide clinicians with more flexibility. Most often, the stress level threshold varies across individuals, as shown during experimentation. Furthermore, it may also vary between time periods (although this is not investigated in the present study). Finally, clinicians at times follow different investigation use cases. They may want to have a quick overview of a large period in time or go in-depth and investigate stress fluctuations in a large resolution, such as per minute or per hour. In order to support all of these scenarios and flexibility, the web application which exposes stress measurements to the clinicians, provides many functions.

**Figure 7:**
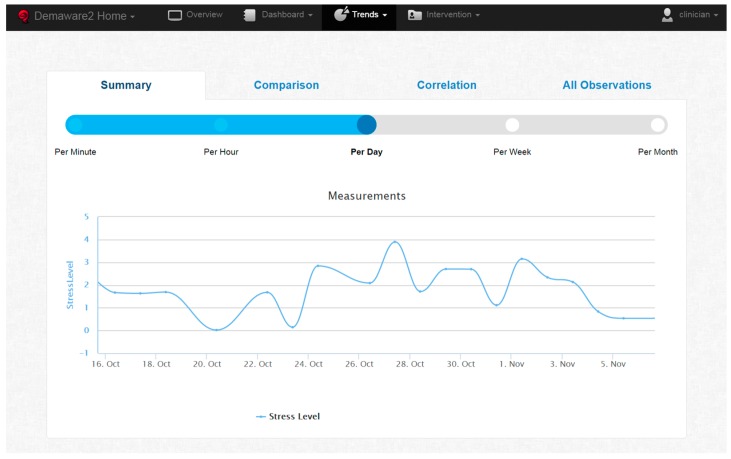
Web application showing daily average stress level.

As shown in Figure 7, it initially supports a slider to pick sampling resolution, ranging from per minute to per month averages for the metrics shown. Then, the general view scenario is supported by showing a line chart of stress level averages, for the selected period and scale. As Figure 7 shows, daily average stress level fluctuations are presented as measured for the clinician to be able to investigate the patterns themselves.

**Figure 8:**
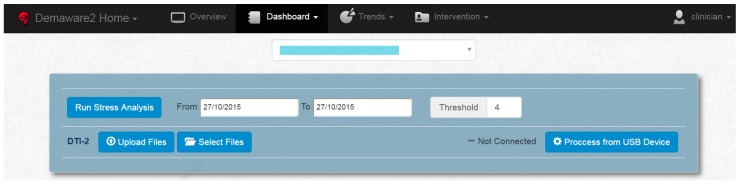
Dashboard for the clinicians to run analysis, setting a personalized stress level threshold (“Threshold”) for each user (anonymized drop-down menu) and time period (“From”–“To” fields).

Alternatively, the web application also serves for personalized investigations of stress fluctuations as follows. The Dashboard, shown on Figure 8, allows a clinician to run a stress analysis, namely set a stress threshold for a given individual (anonymized in the figure) and a given time period. This simply marks “High Stress” events in the log of measurements where they exceed the given, personalized threshold.

**Figure 9:**
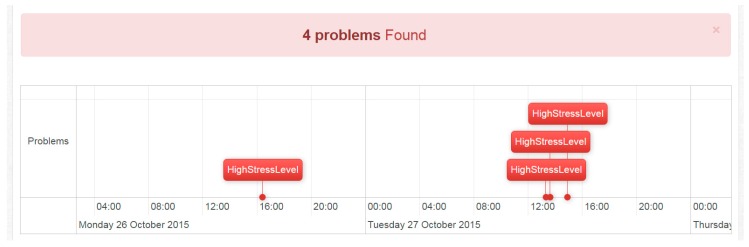
View of High Stress level problems, according to personalized thresholds.

The result of the stress analysis is shown on Figure 9. A timeline of stress events is displayed, essentially hiding all detailed stress level measurements from the clinician. This view is particularly useful when reviewing an individual’s log at a glance. Stress level threshold and period combinations may be changed at any time.

## 6. Conclusions

This paper presented a framework to measure and highlight stress events and also presented it as a sign of behavioral patterns of people with dementia to clinical nursing home staff. The system utilizes a wearable sensor that measures GSR. GSR data is processed using a per-user trained statistical method, extracting stress measurements classified in five levels of severity. The method was evaluated by six participants wearing the equipment over a period of two months with thirty staff members taking clinical notes to serve as ground truth. Experimenting with stress level thresholds showed that different thresholds can serve different scenarios. For instance, a high threshold yields high accuracy and high precision, a low threshold yields high recall, while the medium threshold achieves the best balance between them. This stimulates research for more tailored prediction methods based on machine learning.

Future work will focus on collecting more data from real users to further validate the presented method in this article. A pilot is currently running with a new wristband, called Empatica E4 [33], which measures the GSR level, accelerometers, and pulses. The data from the new sensor will be used to further develop the stress detection algorithm to approach better results. Pulse measurements, for instance, will be integrated into the algorithm to provide better detection of stress episodes and to achieve the best fit per scenario and even per user.

## Figures and Tables

**Table 1:** Comparison of the related work.

Authors	Sensors	Analysis	Application Domain
(Setz et al., 2010)	Wearable electrodermal activity (EDA) device.	Calculate the stress level from the EDA data	Personal health system at the workplace
(Zhai et al., 2006)	Sensors placed on the hand: galvanic skin response (GSR), blood volume pulse (BVP), skin temperature (ST), and eye gaze tracking instrument: pupil diameter (PD)	Calculate the stress level from the collected data	Emotion recognition system
(Hosseini et al., 2010)	Flexcom Infiniti biofeedback device: skin conductance (SC), photoplethysmograph (PPG), respiratory rate (RR) and EEG.	Calculate the stress level by analyzing multi-modal bio-signals	Emotional stress recognition system
(Kirschbaum et al., 1989)	N/A	Calculate the stress level from the cortisol level in saliva	N/A
(Lupien, 2013)	Simple vital signs monitor	Calculate the stress level from the blood pressure	N/A
(Villarejo et al., 2012)	Two electrodes placed on the fingers: Galvanic Skin Response (GSR)	Calculate the stress level based on GSR data	Stress monitoring system
(Bakker et al., 2011)	Watch-style stress measurement device: Galvanic Skin Response (GSR)	Calculate the stress level based on GSR data	Stress management system
(Perala et al., 2007)	Armband worn on the back of the upper arm: Galvanic Skin Response (GSR)	Calculate the stress level based on GSR data	Stress monitoring system
(Hernandez et al., 2011)	Wrist skin conductance sensor: Galvanic Skin Response (GSR)	Calculate the stress level based on GSR data	Stress monitoring system
(Sarker et al., 2016)	Chest-worn band (Accelerometer, respiration, electrocardiogram (ECG))	Predict significant stress episodes from time-series data	Just-in-time interventions at work or daily life
(Gjoreski et al., 2015)	Smartphone: Accelerometer, Audio (ambient noise), Wi-Fi, Call logs, Battery level, Light	Assess student behavior from sensors and questionnaires	School student stress assessment
(Vic Rodney., 2000)	N/A	Calculate the stress/aggression level based a checklist of questions	Stress monitoring system, Dementia Care
(Mackay et al., 1978)	N/A	Calculate the stress/aggression level based on a checklist of questions	Stress monitoring system, Dementia Care
(Vedhara et al., 1999)	N/A	Calculate the stress using Global Measure of Perceived Stress scale	Dementia Care
(Cohen et al., 1983)	N/A	Calculate the stress using Global Measure of Perceived Stress scale	N/A
(Algase et al., 2003)	Step watch	Assess the wandering behavior, and use it as a sign for stress	Stress monitoring system, Dementia Care
Our Approach	Wristband sensor: Galvanic Skin Response (GSR), accelerometers data (ACC)	Calculate the stress level based on GSR and ACC data	Stress monitoring system, Context management, User profiling, Dementia Care

**Table 2:** Observation note instances per user, showing the positive and negative count, and positive instances percentage to total.

User	Positive (Stressed)	Negative (Not Stressed)	User Total	Positive/User Total
User 1	42	464	506	8.30%
User 2	8	379	387	2.07%
User 3	29	193	222	13.06%
User 4	41	223	264	15.53%
User 5	101	254	355	28.45%
User 6	19	689	708	2.68%
All Users	240	2202	2442	9.83%

**Table 3:** Information schema for representing ground truth from clinical notes and analyzed sensor input.

Field	Person ID	Start Time	End Time	Type	Provider
Type	String	Datetime as a UNIX Timestamp	Datetime as a UNIX Timestamp	‘Not Stressed’ or ‘Stressed’	‘Clinical Notes’ or ‘Sensor’

**Table 4:** Performance metrics per stress level threshold.

Metric/Stress Level Threshold	0	1	2	3	4	5
Precision	9.9%	12.1%	13.9%	19.1%	20.9%	4.2%
Recall	100.0%	81.7%	65.9%	48.2%	20.4%	0.9%
Accuracy	9.9%	43.3%	59.8%	75.9%	85.0%	89.4%
F-measure	17.6%	20.7%	22.6%	26.8%	18.9%	1.4%
